# Informant Effect on Functional Activities Questionnaire scores in the National Alzheimer's Coordinating Center Uniform Data Set

**DOI:** 10.1002/alz70857_098603

**Published:** 2025-12-24

**Authors:** Juan‐Camilo Vargas‐González, Martin Ingelsson, David F. Tang‐Wai, Carmela Tartaglia

**Affiliations:** ^1^ University Health Network, Toronto, ON, Canada; ^2^ Memory Clinic, Toronto Western Hospital, University Health Network, Toronto, ON, Canada; ^3^ Temerty Faculty of Medicine, University of Toronto, Toronto, ON, Canada; ^4^ University of Toronto, Toronto, ON, Canada

## Abstract

**Background:**

The Functional Activities Questionnaire (FAQ) is one of the most frequently used functionality assessments in patients with Alzheimer's disease (AD). It is an informant‐based assessment wherein informants are asked to rate daily instrumental activities performance in the patient. We have found that informant characteristics influence the Clinical Dementia Rating (CDR) in the National Alzheimer's Coordinating Center Uniform Data Set (NACC‐UDS). We aimed to evaluate informant characteristics' effect on the FAQ in patients with mild cognitive impairment or dementia due to AD participating in the NACC‐UDS.

**Method:**

We included all participants from the NACC‐UDS that had AD as the principal diagnosis, and the Mini‐Mental State Examination or Montreal Cognitive Assessment scores, informant characteristics, CDR global score (CDR‐GS) and the FAQ score. We performed a conditional growth model using multilevel linear regression analysis.

**Result:**

We included 19863 participants, totalling 45596 visits with a median of 2 (1‐4) visits. The patient's age at the initial visit was 74.1±9.4 years and 53.8% were females. Informants were 66.2±13.2 years, 69.2% were females, and the relationship was 61.0% spouse or partner, 26.7% children and 12.3% other.

The FAQ scores were affected by informant characteristics. The FAQ scores were 0.48 (CI 95%:0.31 to 0.65) higher with female informants, 2.04 (CI 95%: 1.79 to 2.28) higher when informants were children, and 0.30 (CI 95%: 0.01 to 0.60) higher when informants were other family members, both compared with spouses or partners. The frequency of visits was associated with a FAQ score 0.57 lower (CI 95%:‐0.89 to ‐0.26) when visiting daily, 1.54 lower (CI 95%:‐1.78 to ‐1.29) when visiting at least once per week, and 2.50 lower (CI 95%:‐2.80 to ‐2.20) when visiting less than once a week compared with living with the patient. As expected, FAQ scores increased with lower Z‐standardized cognitive scores.

**Conclusion:**

We found that the FAQ scores are modified by informant characteristics in the NACC‐UDS patients with AD diagnosis. These results are clinically relevant because FAQ is often used to evaluate the severity of AD impact on instrumental activities of daily and monitor the functional decline in AD.

